# Evidence-based diagnosis and treatment of macrophage activation syndrome in systemic juvenile idiopathic arthritis

**DOI:** 10.1186/s12969-015-0055-3

**Published:** 2015-12-03

**Authors:** V. Boom, J. Anton, P. Lahdenne, P. Quartier, A. Ravelli, N.M. Wulffraat, S.J. Vastert

**Affiliations:** Department of Pediatric Rheumatology, Wilhelmina Children’s Hospital, University Medical Center Utrecht, Utrecht, The Netherlands; Pediatric Rheumatology Unit, Hospital Sant Joan de Déu. Universitat de Barcelona, Barcelona, Spain; Department of Pediatrics, Children’s Hospital, University Hospital Helsinki, Helsinki, Finland; Pediatric Immunology-Hematology and Rheumatology Unit and IMAGINE Institute, Necker-Enfants Malades Hospital, Assistance Publique Hopitaux de Paris and Universite Paris-Descartes, Paris, France; Department of pediatric rheumatology, Instituto Giannini Gaslini, Genua, Italy

**Keywords:** SJIA, MAS, Hemophagocytosis, Systemic, Arthritis, Complications, Diagnosis, Treatment, Biomarkers, HLH

## Abstract

**Background:**

Macrophage activation syndrome (MAS) is a severe and potentially lethal complication of several inflammatory diseases but seems particularly linked to systemic juvenile idiopathic arthritis (sJIA). Standardized diagnostic and treatment guidelines for MAS in sJIA are currently lacking. The aim of this systematic literature review was to evaluate currently available literature on diagnostic criteria for MAS in sJIA and provide an overview of possible biomarkers for diagnosis, disease activity and treatment response and recent advances in treatment.

**Methods:**

A systematic literature search was performed in MEDLINE, EMBASE and Cochrane. 495 papers were identified. Potentially relevant papers were selected by 3 authors after which full text screening was performed. All selected papers were evaluated by at least two independent experts for validity and level of evidence according to EULAR guidelines.

**Results:**

27 papers were included: 7 on diagnosis, 9 on biomarkers and 11 on treatment. Systematic review of the literature confirmed that there are no validated diagnostic criteria for MAS in sJIA. The preliminary Ravelli criteria, with the addition of ferritin, performed well in a large retrospective case-control study. Recently, an international consortium lead by PRINTO proposed a new set of diagnostic criteria able to distinguish MAS from active sJIA and/or infection with superior performance.

Other promising diagnostic biomarkers potentially distinguish MAS complicating sJIA from primary and virus-associated hemophagocytic lymphohistiocytosis.

The highest level of evidence for treatment comes from case-series. High dose corticosteroids with or without cyclosporine A were frequently reported as first-line therapy. From the newer treatment modalities, promising responses have been reported with anakinra.

**Conclusion:**

MAS in sJIA seems to be diagnosed best by the recently proposed PRINTO criteria, although prospective validation is needed. Novel promising biomarkers for sJIA related MAS are in need of prospective validation as well, and are not widely available yet. Currently, treatment of MAS in sJIA relies more on experience than evidence based medicine. Taking into account the severity of MAS and the scarcity of evidence, early expert consultation is recommended as soon as MAS is suspected.

## Background

Macrophage activation syndrome (MAS) is an intriguing and potentially life-threatening condition [1, 2], clinically characterized by non-remitting fever, hepatosplenomegaly, lymphadenopathy, encephalopathy, coagulopathy and even multi organ failure in severe cases. Laboratory abnormalities of MAS include pancytopenia, hyperferritinemia, hypertriglyceridemia and elevated serum transaminases [3]. MAS has been reported to occur in the context of infectious-, malignant-, metabolic- and auto-immune diseases [4] but seems particularly linked to systemic Juvenile Idiopathic Arthritis (sJIA), occurring in at least 7–13 % of sJIA patients [1, 5, 6]. The actual incidence of MAS in sJIA is likely to be even higher, as bone marrow evidence of subclinical MAS was found in more than 53 % of sJIA patients at the time of diagnosis [5].

SJIA is a subtype of Juvenile Idiopathic Arthritis (JIA) and is characterised by arthritis of unknown origin and extra-articular symptoms like spiking fever, often accompanied with a macular rash, serositis, hepatosplenomegaly and generalised lymphadenopathy due to reticuloendothelial involvement. SJIA is considered an (acquired) auto-inflammatory disease rather than an autoimmune disease because of clear clinical and pathophysiological differences when compared to the other subtypes of JIA. Moreover, sJIA lacks clear association with HLA-types or auto-antibodies. Pathophysiologically, it is now clear that mechanisms related to the innate immune system, especially driven by IL-1, IL-6 and IL-18, are pivotal in sJIA [7, 8]. Its disease course can be unpredictable, varying from a monophasic course of relatively mild disease to chronic relapsing periods of severe poly-arthritis accompanied by critical extra-articular symptoms and complications causing significant morbidity and mortality.

Nowadays, MAS in sJIA is considered an acquired or secondary hemophagocytic lymphohistiocytic (HLH) disorder [9]. In primary HLH, defective control of T cell activation, including in many cases defects in the NK cell cytolytic pathway, underlies uncontrolled cytokine production resulting in excessive activation and tissue invasion of T lymphocytes and macrophages. The enormous cytokine storm and blood cell hemophagocytosis by CD163^+^ macrophages are likewise responsible for the clinical and laboratory features of HLH and resemble those of MAS in sJIA [4]. Moreover, the strong association of MAS with sJIA hints to shared pathophysiological mechanisms with the other HLH-syndromes. Diagnostic guidelines for (acquired) HLH are available (HLH-2004) [10] which are sometimes used for diagnosis of MAS in sJIA.

Diagnosis of MAS in sJIA patients can be challenging since MAS is difficult to distinguish from a flare of sJIA or from sepsis [1, 6, 11] and certain treatments of sJIA, such as tocilizumab, can conceivably mask the clinical and biologic features of MAS,[12]. Moreover, treatment is generally based on the practitioner's experience. The aim of this systematic literature review was therefore to evaluate published sets of diagnostic criteria for MAS in sJIA and provide an overview of possible biomarkers for diagnosis, disease activity and treatment response. In addition, the literature was searched for published data on treatment of MAS.

## Methods

A first systematic literature search was conducted on the 1^st^ of June 2014, comprising all English articles from 1970 onwards in the MEDLINE, EMBASE and Cochrane databases. MEDLINE was searched through Pubmed by searching for the medical subject headings (MeSH) (Arthritis, Juvenile Rheumatoid), (Lymphohistiocytosis, Hemophagocytic) and (Macrophage Activation Syndrome) supplemented with the keywords JIA, JRA, Still’s disease, MAS, lymphohistiocytosis and synonyms. For EMBASE, MeSH terms were replaced by the corresponding Emtree terms. The Cochrane Central Register of Controlled Trials (CENTRAL) was searched for the same keywords. Search strings were built under supervision of specialized librarians and validated for completeness by performing cited reference checks (www.scopus.com). Exclusion criteria were adult studies, case-reports, case-series containing less than 3 cases, meeting abstracts, reviews and articles not considering diagnosis, biomarkers or therapy of MAS in sJIA. Included articles were initially selected based on title and abstract by two authors separately, after which full text screening was performed. For every paper, the category of evidence was determined by two reviewers according to EULAR guidelines [13]. In case of disagreement on in- or exclusion of a paper or category of evidence, the full text was discussed in the team to reach consensus. During the review process of this paper a second literature search was conducted on September 22^nd^, 2015 allowing inclusion of the most recently published papers. The search strategy and inclusion methods were identical to the first search. The category of evidence of all papers additionally included in the second search was evaluated by two authors (SV and NW).

## Results

**Fig. 1:**
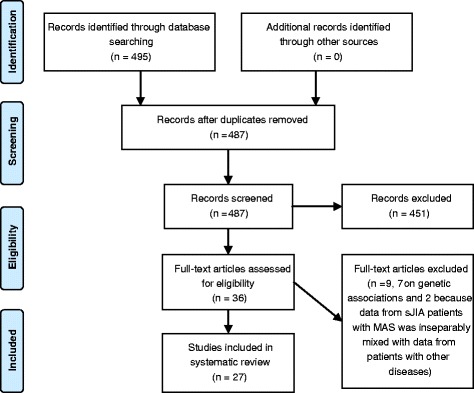
Flow chart of the systematic review process

A total of 495 potentially relevant titles were identified through two literature searches, of which 36 papers were retrieved for full text screening (Fig. 1). In total, we included 27 eligible papers: 7 on diagnosis, 9 on biomarkers and 11 on treatment [Table 1]. The level of evidence (LOE) of included papers is listed in Table 1. Nine papers were excluded after full text screening. Two papers on biomarkers were excluded because data of sJIA patients with MAS were inseparably mixed with MAS in the context of other underlying diseases [14, 15]. 7 papers assessing genetic associations of MAS in sJIA were excluded because these papers addressed possible genetic associations as possible underlying causes rather than as possible biomarkers for diagnosis, disease activity or treatment response. Of note, one paper describing disease features as well as treatments used worldwide [16] was only included in the diagnosis section of this review, since specific information on (effectivity of) therapeutic regimens used is not listed.

**Table 1:** Included papers on diagnosis, biomarkers and treatment of MAS in sJIA patients

Authors	Year	Number of sJIA patients with MAS	Subject	Content	Evaluation design	Results	Conclusion	Limitations	LOE [13]
Ravelli et al.[3]	2005	74	diagnosis	Diagnostic criteria of MAS	Comparative study	A set of preliminary clinical and laboratory criteria of MAS in sJIA.	Preliminary Ravelli criteria [Table 3].	Not validated, lacks ferritin as parameter.	3
Davi et al.[18]	2014	362	diagnosis	Assessment of performance of diagnostic guidelines	Retrospective study	The preliminary Ravelli criteria perform better than the HLH-2004 guidelines in differentiating MAS from active sJIA or infection (by adding ferritin as parameter).	The preliminary diagnostic criteria perform better than the HLH-2004 guidelines.	Retrospective study design, selection bias	3
Minoia et al.[16]	2014	362	diagnosis	Disease features of MAS in sJIA	Descriptive study	Decreased platelet counts and increased ASAT, triglycerides, ferritin and LDH levels were the most common laboratory features during onset of MAS. Fever and organomegaly were the most frequent clinical symptoms.	The clinical spectrum of MAS in sJIA comprises frequently reported clinical and laboratory features.	Retrospectively collected data, possible selection bias.	3
Minoia et al.[19]	2015	362	diagnosis	Clinical heterogeneity of MAS in sJIA	Descriptive study	Clinical and laboratory features of MAS in sJIA did not differ among patients registered from different geographic locations.	The clinical spectrum of MAS is comparable across patients from different geographic locations.	Retrospectively collected data, possible selection bias.	3
Ravelli et al.[21]	2015	362	diagnosis	Cross-validated literature- and consensus based diagnostic guidelines for MAS in sJIA	Comparative study complemented with expert opinion	A final set of diagnostic (laboratory) criteria was approved based on the selection of best classification criteria through statistical analyses and consensus formation techniques with a higher sensitivity and specificity compared to the preliminary Ravelli criteria.	Best performing set of diagnostic criteria for MAS in sJIA [Table 3].	Not prospectively validated, level of evidence low due to incorporation of expert opinion.	3/4
Kostik et al.[22]	2015	18	diagnosis	Diagnostic criteria	Comparative study	Laboratory criteria were more precise in discriminating MAS from active sJIA than clinical variables. Eight widely available laboratory markers were selected as best for early identification of MAS.	Preliminary diagnostic criteria.	Retrospective, no evaluation of changes in laboratory parameters.	3
Lehmberg et al.[23]	2013	27	diagnosis	Differentiating MAS in sJIA from HLH	Retrospective study	Generally available laboratory measures with accessory cut-off values to distinguish MAS complicating sJIA from primary HLH and virus-associated HLH (VA-HLH) were retrospectively identified.	Neutrophil counts >1.8 x 10^9^/L, CRP >90 mg/L and sCD25 <7900 U/ml indicate MAS in sJIA rather than primary HLH or VA-HLH.	No control group, no cut-off points.	3
Grom et al.[24]	2002	7	biomarkers	NK cell function	Comparative study	NK cell activity was decreased in all patients compared to healthy controls. Low NK cell activity was associated with decreased numbers of NK cells.	NK dysfunction is common in sJIA associated MAS	Small patient sample.	3
Bleesing et al.[25]	2007	7	biomarkers	sCD25, sCD163	Comparative study	sCD25 and sCD163 were significantly higher in the acute phase of MAS compared to untreated new-onset sJIA patients and correlated with disease activity.	sCD35 and sCD163 are promising biomarkers of MAS	Small number of patients, not validated	3
Reddy et al.[26]	2014	2	biomarkers	sCD25, sCD163 as markers of subclinical MAS in active sJIA	Comparative study	Laboratory abnormalities associated with MAS were seen in active sJIA patients with elevated levels of sCD25 and to a lesser extend in patients with elevated levels of sCD163.	sCD25 might be a marker of subclinical MAS in active sJIA.	Only 2 MAS patients, not validated	3
Gorelic et al.[27]	2013	7	biomarkers	FSTL-1, ferritin/ESR ratio	Comparative study	FSTL-1 levels during MAS are elevated compared to active sJIA. Elevated levels of FSTL-1 were associated with occult MAS, correlated with levels of sCD25 and ferritin and normalized after treatment. Ferritin/ESR ratio was superior to ferritin in discriminating MAS from new-onset sJIA.	Elevated levels of FSTL-1 might be a marker of occult MAS.	FSTL-1 is unspecific, small sample size, not validated	3
Shimizu et al.[8]	2010	5	biomarkers	IL-6, IL-18, neopterin for differentiating MAS in sJIA from VA-HLH or KD	Comparative study	IL-18 was significantly higher in MAS in sJIA compared to EBV-HLH or KD and correlated with measures of disease activity. IL-6 was higher in KD patients and neopterin was higher in EBV-HLH.	Serum cytokine profiles differ between MAS in sJIA, KD and EBV-HLH. IL-18 might be useful for differentiation of MAS in sJIA from HLH.	Small sample, not validated	3
Shimizu et al.[12]	2012	5	biomarkers	IL-18, IL-6 during TCZ treatment	Comparative study	TCZ can suppress clinical symptoms of MAS. IL-18 and IL-6 were elevated during MAS in patients with and without TCZ and correlated with disease activity.	During TCZ treatment, monitoring IL-18 and IL-6 could be useful to disclose early MAS.	Only 5 MAS patients, not validated	3
Yokota et al.[28]	2015	14	biomarkers	Changes in laboratory markers in patients with MAS receiving TCZ	Retrospective Descriptive study	Most patients had common laboratory features associated with MAS.	Clinical and laboratory features of MAS appear similar among patients with and without TCZ treatment.	No control group, retrospective	3
Shimizu et al.[29]	2015	15	biomarkers	Serum IL-18 as biomarker for the prediction of MAS in sJIA	Comparative study	During active sJIA, IL-18 levels >47750 pg/ml predicted development of MAS. IL-6 levels in patients with MAS did not differ from IL-6 levels during active sJIA in absence of MAS.	Serum IL-18 levels > 47750 pg/ml might be a biomarker for MAS development	High cut-off values suggest low sensitivity	3
Kounami et al.[30]	2005	5	biomarkers	urine β2-microglobulin	Descriptive study	Urinary β2-microglobulin levels increased during MAS.	Increases in urinary β2-microglobuline might be an indicator of MAS.	No control group, small sample size, not specific	3
Sawhney et al.[6]	2001	8	treatment	steroids, CsA, eto	Case-series	Patients received steroids as part of a combinational regimen, of which >62% in combination with CsA.	High dose steroids in combination with CsA was effective in cases of MAS.	Small retrospective case-series	3
Mouy et al.[35]	1996	5	treatment	steroids, CsA	Case-series	CsA monotherapy was effective in 7 episodes of MAS and was effective in 3 episodes of steroid-resistant MAS.	CsA can be effective as first or second line (mono) therapy.	Small retrospective case-series	3
Stephan et al.[1]	2001	18	treatment	steroids, CsA, IVIG, eto	Case-series	CsA as initial monotherapy induced remission in 5 cases. CsA was effective in 6 cases of steroid-resistant MAS. Steroids were effective as first-line (mono) therapy. IVIG was not effective.	CsA and steroids were effective as first-line monotherapy or combined.	Small retrospective case-series	3
Miettunen et al.[37]	2011	8	treatment	Anakinra	Case-series	Anakinra was effective in 8 cases of conventional therapy- resistant MAS.	Anakinra was effective in cases where initial therapy with steroids and CsA failed.	Small retrospective case-series	3
Ramanan et al.[31]	2004	3	treatment	(pulse) steroids, eto	Case-series	Steroid monotherapy was effective in 3 patients with MAS with renal involvement.	Steroids can be effective as monotherapy in patients with renal involvement complicating MAS.	Small retrospective case-series	3
Lin et al.[2]	2012	4	treatment	steroids, IVIG, CsA	Case-series	Prednisolone was effective as monotherapy or in combination with CsA. IVIG was not effective.	Patients responded well to steroids and CsA.	Small retrospective case-series	3
Kounami et al.[30]	2005	5	treatment	steroids, IVIG, CsA	Case-series	All patients treated with CsA as first or second line therapy responded well. IVIG failed as first-line treatment.	CsA was effective as first-line (mono) therapy.	Small retrospective case-series	3
Singh et al.[11]	2011	6	treatment	steroids, IVIG	Case-series	Four patients responded to high dose methylprednisolone, 1 patient recovered after addition of IVIG to steroids.	Steroids were effective as initial monotherapy.	Small retrospective case-series	3
Cortis et al.[36]	2006	9	treatment	steroids, CsA, etanercept	Case-series	7 cases of MAS responded to high dose steroids with or without CsA. In one patient, a third episode of MAS responded to etanercept when steroids and CsA failed.	Patients responded well to steroids and CsA.	Small retrospective case-series	3
Zeng et al.[32]	2008	13	treatment	steroids, eto, VCR, IVIG	Case-series	Steroids were effective as first-line (mono) therapy. 1 patient responded to eto after steroids, CsA and IVIG failed.	Steroids were effective as first-line (mono) therapy.	Small retrospective case-series	3
Nakagishi et al.[34]	2014	3	treatment	Dexamethasone palmitate	Case-series	All three patients were resistant to methylprednisolone but responded well to dexamethasone palmitate.	DexP can be effective in mps-resistant MAS.	Small case-series	3

Abbreviations: *LOE* level of evidence; *MAS* Macrophage Activation Syndrome; *sJIA* systemic juvenile idiopathic arthritis; ASAT aspartate aminotransferase; LDH lactate dehydrogenase; *HLH* hemophagocytic lymphohistiocytosis; CRP C-reactive protein; *VA-HLH* virus-associated hemophagocytic lymphohistiocytosis; *FSTL-1* Follistatin-related protein 1; ESR erythrocyte sedimentation rate; *EBV-HLH* Epstein-Barr related hemophagocytic lymphohistiocytosis; *KD* Kawasaki disease; *TCZ* Tocilizumab; *CsA* cyclosporine A; *VCR* vincristine; *IVIG* intravenous immunoglobulin; eto etoposide; *DexP* Dexamethasone palmitate

### Diagnosis of MAS in sJIA

Seven papers were found on diagnostic criteria for MAS in sJIA [Table 1]. The level of evidence of the included papers was 3, except when listed otherwise [Table 1]. In 2005, Ravelli et al. proposed preliminary diagnostic guidelines based on clinical and laboratory parameters, established to differentiate between MAS and flare of active sJIA [3]. The combination of variables with the highest ability to distinguish between disease and controls was obtained by a statistical approach [17]. Presence of any 2 or more laboratory or of any 2 or more clinical and/or laboratory criteria was required for the diagnosis of MAS [Table 2].

**Table 2:** Preliminary diagnostic guidelines for MAS complicating sJIA [3]

Clinical criteria	
1. Central nervous system dysfunction	
2. Haemorrhages	
3. Hepatomegaly	
Laboratory criteria	
1. Decreased platelets count (≤262 × 10^9^/L)	
2. Elevated levels of aspartate aminotransferase (>59 U/L)	
3. Decreased white blood cell count (≤4.0 × 10^9^/L)	
4. Hypofibrinogenemia (≤2.5 g/L)	

Presence of any 2 or more laboratory or of any 2 or more clinical and/or laboratory criteria is required for the diagnosis of MAS in sJIA

The performance of the preliminary diagnostic criteria by Ravelli was assessed by Davi and co-workers, in a multinational initiative lead by the Pediatric Rheumatology International Trials Organization (PRINTO). This collaboration evaluated a large retrospective cohort of sJIA patients with MAS (362 patients), sepsis (345 patients) and active sJIA patients without MAS (404 patients) and compared the performance of the preliminary Ravelli criteria to the HLH-2004 diagnostic guidelines to differentiate MAS in sJIA from a flare of sJIA and sepsis [18]. Within this cohort, the preliminary diagnostic criteria performed best at differentiating MAS in sJIA from a flare of sJIA (sensitivity 86 %, specificity 86 %, κ 0.71) or infection (by adding hyperferritinemia (≥500 ng/ml) as a parameter (sensitivity 86 %, specificity 95 %, κ 0.76)).

In continuation of this collaboration, Minoia et al compared clinical and laboratory parameters of the 362 patients with sJIA associated MAS [16]. More than 90 % of patients showed decreased platelet counts and increased aspartate aminotransferase, triglycerides, ferritin and lactate dehydrogenase levels during onset of MAS [16]. Importantly, serum ferritin was the laboratory marker showing the largest change in pre-MAS and MAS-onset values. This group also showed that laboratory features of MAS were comparable across patients registered from different geographic locations [19].

Importantly, the PRINTO collaboration also aimed to develop a new set of diagnostic criteria through a multistep process. First, candidate diagnostic criteria were identified from a questionnaire, listing 28 clinical, laboratory and histopathological features that was sent to 505 paediatric rheumatologists worldwide [20]. Partly based upon the results of this questionnaire, a new set of (laboratory) diagnostic criteria was proposed by PRINTO in 2015 to distinguish MAS in sJIA from MAS imitating conditions (LOE 3–4) [21]. Expert consensus procedures were carried out for distinguishing MAS in sJIA from non-MAS whereupon expert consensus was considered as diagnostic ‘golden standard’. A final set of diagnostic criteria was approved based on the selection of best classification criteria through statistical analyses and consensus formation techniques with an 82 % consensus among 28 international experts. These laboratory parameters are listed in Table 3. Cross-validation of these criteria was performed by comparing the performance of these criteria to the expert opinion ‘golden standard’ in the entire patients database of 1.111 patients and showed a sensitivity of 0.72, a specificity of 0.97, a positive predictive value of 93.9 %, a negative predictive value of 84.8 %, an area under the curve of 0.84 and a kappa for agreement of 0.72.

**Table 3:** PRINTO diagnostic criteria for MAS in sJIA [21]

A patient with (suspected) sJIA with	
Fever and serum ferritin > 684 ng/ml	
AND any 2 of the following	
Platelet count ≤181 × 10^9^/L	
Aspartate aminotransferase (>48 U/L	
Triglycerides > 156 mg/dl	
Fibrinogen ≤360 mg/dl	

Laboratory abnormalities should not be otherwise explained by the patient’s condition, such as concomitant immune-mediated thrombocytopenia, infectious hepatitis, visceral leishmaniasis, or familial hyperlipidemia

Also Kostik et al. created preliminary diagnostic guidelines for early discrimination of MAS in patients with active sJIA [22]. Clinical and laboratory features of 18 patients with active sJIA with MAS and 40 patients with active sJIA without MAS were reviewed and compared retrospectively. Laboratory criteria were more precise in discriminating MAS from active sJIA than clinical variables. Eight widely available laboratory markers with cut-off points were selected as best for early identification of MAS: white blood cell counts ≤9.9 × 109/L, platelet counts ≤211 × 109/L, aspartate aminotransferase >59.7 U/L, lactate dehydrogenase > 822 U/L, albumin <29 g/L, ferritin >400 μg/L, fibrinogen ≤1.8 g/L and the presence of proteinuria. Presence of 3 and more criteria provided the highest specificity and sensitivity (1.0, 1.0) with the highest odds ratio (2997 (57-156963) in the whole model.

MAS can be the presenting feature of sJIA even prior to onset of characteristic symptoms like arthritis. In 2013, Lehmberg et al. retrospectively identified generally available laboratory measures with accessory cut-off values to distinguish MAS complicating sJIA from primary HLH and virus-associated HLH (VA-HLH) [23]. Data from sJIA patients that presented with MAS as initial feature of sJIA was obtained from the German national HLH study centre. Neutrophil counts >1.8 × 10^9^/L (sensitivity 85 %, specificity 83 %), CRP >90 mg/L (74 %, 89 %) and sCD25 < 7900 U/ml (79 %, 76 %) indicated MAS in sJIA rather than primary HLH or VA-HLH.

### Novel biomarkers of MAS in sJIA

For this review, we identified 9 eligible studies on promising biomarkers for MAS in sJIA. All papers were category of evidence 3 [Table 1] and studied 1 or more (inflammatory) biomarkers for diagnostic potential in sJIA related MAS.

Grom et al. assessed NK cell function in 7 sJIA patients with MAS by a standard ^51^Cr-release assay [24]. NK cell activity was decreased in all patients compared to healthy controls. Furthermore, low NK cell activity was associated with decreased numbers of NK cells.

Bleesing et al. demonstrated soluble IL-2 receptor α chain (sIL-Rα, or sCD25) and soluble CD163 (sCD163) to be significantly higher in the acute phase of MAS compared to untreated new-onset sJIA patients [25]. Moreover, sCD25 and sCD163 levels were found to correlate with disease activity. In a subgroup of patients (*n* = 3) CD163 could also be stained in increased levels in bone marrow specimens. Within their population of 7 sJIA patients with MAS and 16 new-onset sJIA patients without MAS, they identified a subgroup of new-onset sJIA patients with elevation of at least one of these markers to levels associated with overt MAS. Interestingly, these patients had significantly higher levels of ferritin, normal platelet counts, lower haemoglobin levels, and lower NK cell function compared to the other new-onset patients. As these features resemble features of MAS, it was suggested that assessment of sCD25 and sCD163 levels could identify subclinical MAS.

Reddy et al. recently assessed levels of sCD25 and sCD163 in active sJIA patients (*n* = 33) and 2 sJIA patients with MAS [26]. They found multiple laboratory abnormalities suggestive of MAS in their patient group with the highest sCD25 and sCD163 levels. In contrast to sCD163 levels, sCD25 levels correlated significantly with CRP, Hb and LDH.

Gorelik et al. reported Follistatin-related protein 1 (FSTL-1) levels to be elevated in active sJIA and to be even more elevated during MAS [27]. In their cohort of 27 sJIA patients including 7 patients with MAS, elevated levels of FSTL-1 were associated with occult MAS, correlated with levels of sCD25 and ferritin and normalized after treatment. In addition, in an effort to find biomarkers distinguishing between MAS and new-onset sJIA, a ferritin to ESR ratio > 80 had the highest sensitivity and specificity (100 %, 100 %) in their cohort.

Shimizu et al. studied cytokines during MAS in serum of sJIA patients and compared them to cytokine patterns in EBV-induced HLH (EBV-HLH), Kawasaki disease (KD) and healthy age matched controls [8] IL-18 concentrations during MAS were significantly higher compared to EBV-HLH and KD and were found to correlate to measures of disease activity (CRP, ferritin, LDH and other cytokines). In addition, serum neopterin and sTNF-RII levels were significantly higher during MAS compared to flares of sJIA. IL-6 concentrations in patients with KD were significantly higher compared to EBV-HLH or MAS patients, whereas high neopterin concentrations hinted toward EBV-HLH rather than MAS or KD.

In a subsequent study following up on this, Shimizu et al. studied IL-18 and IL-6 for MAS in sJIA during Tocilizumab (TCZ) treatment [12]. Although TCZ was able to suppress clinical symptoms of active sJIA and MAS, IL-18 and IL-6 were consistently elevated during periods of MAS. Moreover, both in patients with and without TCZ, IL-18 and IL-6 levels correlated with disease activity and increased consistently before elevation of other inflammatory parameters. This suggests that during TCZ treatment, monitoring these cytokines could be useful to disclose MAS in an early phase, possibly before onset of overt clinical symptoms.

Yokota et al. studied the 25 cases of MAS that were reported in a phase IV registry of sJIA patients receiving TCZ in Japan [28]. One aim of this study was to confirm changes in laboratory markers suggestive of MAS in patients with MAS during TCZ treatment. Diagnosis of MAS was re-evaluated in every patient on the basis of reported clinical and laboratory data by a committee comprised of 2 pediatric rheumatologists, 1 pediatric specialist in infectious diseases, 1 pediatric cardiologist and 1 adult rheumatologist. 15 cases were considered as definite or probable MAS, 2 cases as EBV-HLH and 8 cases as possible MAS or non-MAS. Although not specified, the authors report that common clinical and laboratory findings suggestive of MAS were observed in the definite or probable MAS group and suggest that clinical and laboratory features (like platelets, liver enzymes, coagulation markers and ferritin) of MAS appear similar among patients with and without TCZ treatment.

In another recent case-control study, Shimizu et al. studied the clinical significance IL-6 and IL-18 in serum of sJIA patients for predicting MAS [29]. Levels of IL-6 and IL-18 were measured in serum of 76 sJIA patients with active disease of which 15 patients developed MAS. During MAS, IL-18 levels were significantly higher compared to patients without MAS. Interestingly, the patients with active sJIA who developed MAS during the course of the disease had significantly higher serum IL-18 levels compared to those who did not develop MAS [29]. During active sJIA, serum IL-18 levels >47750 pg/ml predicted development of MAS (sensitivity 86.7 %, specificity 70.5 %). Serum IL-6 levels in patients with MAS did not differ from IL-6 levels during active sJIA in absence of MAS.

Kounami et al. reported highly elevated levels of β2-microglobulin in serum and urine of sJIA patients during episodes of MAS [30]. Although assessed in a small uncontrolled population, comparative measures pre-MAS and during MAS showed an evident rise in urine β2-microglobulin, even in absence of change of other inflammatory parameters.

### Treatment of MAS in sJIA

Currently, there are no validated evidence based treatment guidelines on MAS in sJIA. However, our literature search identified 11 papers elaborating on treatment of MAS in sJIA. All papers were retrospective clinical case-series, category of evidence 3 [13], comprising 96 episodes of MAS in a total of 78 sJIA patients.

High-dose corticosteroid therapy was frequently reported effective as first-line treatment [1, 2, 6, 11, 30–32]. Thirty-four reported episodes of MAS reached full remission on initial steroid monotherapy [1, 2, 11, 30, 31], with remission rates up to 68 % [11, 32]. Although disease severity was not reported consistently, intravenous pulse therapy seemed to be the regimen of choice in more severe cases of MAS. Steroids were reported safe and effective in patients with renal involvement [6, 31, 33]. In one paper, dexamethasone palmitate, a liposome-incorporated form of dexamethasone was effective in a case of MAS that was resistant to pulse methylprednisolone and as part of a combinational regimen in two other patients [34].

Cyclosporine A (CsA), a T-cell blocking agent that has proven its efficacy in other hystiocytic disorders, was also reported to induce remission in the majority of cases. CsA can be given as monotherapy [1, 30], but in most patients was started as part of a combinational regimen [1, 2, 6, 30, 32, 35]. Importantly, CsA was reported life-saving in serious cases of steroid resistant MAS [35].

Etoposide was used in 8 sJIA patients, in every case as part of a first- or second line combination regimen. Etoposide was able to induce rapid recovery in cases of steroid and CsA resistant MAS [36–38]. In these case-series, no serious adverse events were reported.

In total, 14 patients were treated with intravenous immunoglobulin therapy (IVIG), of which six patients received IVIG as initial monotherapy. Remission on monotherapy was achieved in none. For only 1 patient, addition of IVIG to steroids resulted in remission [11].

Biologicals are increasingly used in the treatment of MAS in sJIA. However, only 2 papers included in this review addressed the use of biolocals [2, 37]. Anti-TNFα therapy was used in two patients with unsatisfactory results [2, 37]. Importantly, Miettunen et al. reported on 12 MAS patients, including 8 sJIA patients, treated with anakinra after insufficient response to steroids, IVIG and CsA. Clinical and laboratorial remission was reached in all patients in absence of any side effects. In addition, control of underlying systemic disease was noted in all patients at follow-up.

## Discussion

MAS in sJIA is an intriguing but potentially life-threatening condition with reported mortality rates of 22–30 % [1, 6]. Therefore, prompt diagnosis and initiation of treatment is of vital importance. Critically reviewing published literature on MAS in sJIA confirms the notion that both diagnosis and treatment still rely more on experience than on evidence based medicine. There are no prospectively validated diagnostic criteria for MAS in sJIA. Since clinical and laboratory characteristics of MAS in sJIA resemble those of primary HLH, diagnostic criteria designed for primary HLH(HLH-2004) [10] have been used in diagnosing acquired forms like MAS [32], however with limitations. For diagnosis of MAS, at least 5 of 8 criteria, comprising more advanced tests that are not routinely performed, need to be present. Also, cut-off values of the HLH protocol fall in the cytopenia range and since sJIA is characterized by high levels of leukocytes and platelets, a relative decrease in these parameters may be more suitable for recognition of early MAS.

The preliminary diagnostic guidelines for MAS in sJIA provided by Ravelli are increasingly used and were proved to perform best in diagnosing MAS in sJIA in a retrospectively evaluated cohort of 362 MAS patients and 749 disease controls [18]. Recently, a multinational collaborative project aimed to develop a new set of diagnostic criteria for MAS in sJIA has just been completed. This has resulted in a new set of widely available laboratory parameters with cut-off values, including ferritin as a parameter, and fever as obligatory clinical parameter. Due to incorporation of expert-opinion in the methodology, the LOE of these criteria was considered 3 to 4. However, considering the enormous dataset and high validity of the methodology that underlie these criteria, plus the fact that these criteria have already shown high performances in a cross-validation process, the authors of this review agree that this set of diagnostic criteria is currently the one that is most useful for current diagnostic purposes, notwithstanding some limitations. These guidelines are still in need for prospective validation. In addition, expert consensus on diagnosis was considered the diagnostic ‘golden-standard’ and the performance of these criteria in patients receiving biologics that potentially influence laboratory parameters should still be determined.

Also Kostik et al. identified a set of discriminating laboratory criteria for early discrimination of MAS in sJIA [22]. Although retrospectively assessed in a relatively small (single centre) population, the performance was superior to both the HLH-2004 protocol and the Ravelli criteria. However, these sets have never been evaluated in the same cohort.

There is no widely accepted single biomarker for the diagnosis and/or disease activity for MAS in sJIA. In general, evaluation of biomarkers is complicated by the fact that there is no validated reference test for MAS in sJIA. However, multiple papers suggested promising and interesting candidate biomarkers. Especially for those patients in which MAS is the presenting symptom of sJIA, or any other inflammatory disorder like SLE, virus-associated HLH etc, biomarkers facilitating diagnosis could be of critical value in clinical practice.

The soluble markers sCD25 and sCD163 are specifically of interest since they both reflect prominent pathophysiological characteristics of MAS. sCD25 reflects the amount of T-cell activity and elevated levels have been reported in several inflammatory conditions [39, 40] Therefore, specificity of this biomarker might be an issue. sCD163 is a scavenger receptor released by alternatively activated (M2) macrophages upon activation [41, 42]. Elevated levels of sCD25 and sCD163 are found in both primary and secondary forms of HLH [5, 10, 23, 30], including in MAS in sJIA [25, 26]. In small cohorts, subgroups of sJIA patients with elevated levels of sCD25 in combination with multiple other laboratory abnormalities suggestive of MAS were identified [26], indicating early immunological derailment prior to the overwhelming cytokine storm in MAS, possibly giving justification for intensification of immunosuppressive therapy in these patients.

Despite features of MAS in sJIA have close resemblance to other (primary or secondary) HLH syndromes serum cytokine patterns appear to differ. IL-18 is elevated in active sJIA and is increasingly used as a biomarker for diagnosis and treatment response. Levels of IL-18 are even further increased during episodes of MAS, as are the levels of neopterin and sTNF-RII which both are believed to reflect immune activation [29, 43, 44]. Importantly, the increase of IL-18 appears to be specific for MAS in sJIA as other secondary HLH syndromes are associated with lower levels of IL-18 [8]. Recently it was shown that IFNγ is also markedly elevated during MAS in sJIA [14]. Currently, a multicentre long-term follow-up study is performed to assess effectivity and safety of an anti-IFNγ antibody as treatment of HLH [45].

Another potential biomarker is FSTL-1, a protein produced by cells of mesenchymal origin [46] that is suggested to be a mediator of innate immune pathways that underlie arthritis in sJIA [47, 48]. This inflammatory protein is highly elevated in synovial fluid and serum of sJIA patients [48] and several auto-immune diseases [46]. During (occult) MAS, FSTL-1 levels are even more elevated compared to active sJIA, indicating that FSTL-1 could be a biomarker for early diagnosis of MAS in sJIA when replicated and validated for specificity in other cohorts.

In primary HLH syndromes, pathogenetic mechanisms have extensively been studied. One consistent and intriguing finding is impaired NK cell cytotoxic function, often secondary to mutations in genes involved in the perforin mediated cytolytic pathway [49–51]. Low NK cell function is increasingly described in sJIA as well, with profoundly depressed cytotoxic activity during episodes of MAS [50, 52]. Similar to mutations in the PRF1, related genes including Munc 13-4, have been described in a subset of sJIA patients [51–53], underlining pathophysiological similarities between primary and secondary HLH like MAS. Better understanding of the relation between NK dysfunction, sJIA pathogenesis and the development of MAS should clarity the value of NK cell function assessment in the diagnosis of MAS and possibly the identification of patients at risk.

β2-microglobulin was the only possible biomarker that was studied in urine. It is part of the HLA molecule and enters the bloodstream after metabolic degradation. During chronic inflammation, serum levels of β2-microglobulin can rise after reaching the renal reabsorption threshold, as has been reported in the active phase of hemophagocytic syndromes and MAS in sJIA [30, 54, 55]. To study its relevance as a biomarker, this should now be replicated in a larger study design including a control group.

As becomes clear from the available literature there is no consensus on how to treat MAS in sJIA. Evidence based guidelines are lacking and no clinical trials have been conducted so far. Most experience is still with high dose of systemic steroids, often combined with other (T cell) immunosuppressive therapy [16]. In the largest existing database of MAS cases in sJIA, almost 98 % of the patients received corticosteroids and 61 % received CsA [16]. Presentation of MAS can be sudden and its course overwhelming. Therefore, physicians must aim for immediate and profound immunosuppression. During MAS, low-dose steroid therapy can be inadequate [1, 35]. In our experience and in accordance with the literature, high dose ‘pulse’ methylprednisolone therapy seems more effective. In addition to steroids, CsA was reported safe and highly effective as first-line monotherapy and has shown its critical value in steroid resistant MAS [35]. Accordingly, CsA plus systemic steroids, seems a fair first choice in the treatment of MAS Of note, CsA does not seem to influence underlying systemic disease and it is unknown for how long the treatment should be continued after clinical improvement. Consequently, flares of MAS have been reported after abrupt termination of CsA [35]. Because of the classification within the HLH syndromes, etoposide, a chemotherapeutic agent, is regularly used as this is part of the first line treatment regimen designed for primary HLH [10]. However, due to possible harmful side-effects, etoposide is by some considered as a last resort.

In the last decade, biologicals are increasingly used as treatment of MAS, however with varying results [56–58]. Based on the included literature, there is insufficient evidence for the use of IVIG or anti-TNFα therapy for MAS in sJIA.

IL-1 blocking therapy with anakinra is increasingly used in MAS as well. In 2011, a case-series was published, reporting on 8 (severe) cases of MAS in sJIA that reached rapid remission on anakinra when conventional therapy failed [37, 59, 60]. In addition, anakinra induced remission of underlying disease and therefore seems advantageous over CsA. Another possible advantage of this more targeted therapy could be a decrease of side effects related to more extensive immunosuppressive therapy. However, anakinra is not available in every country and ideally its effectivity and safety should be compared to the effects of ‘pulse’ steroidal therapy and/or CsA in a randomised setting.

## Conclusion

In conclusion, early diagnosis of MAS in sJIA, and prompt start of treatment is crucial to improve outcome but is often challenging in clinical practice. Recently, consensus was reached on a robust set of widely available diagnostic criteria which scored good performance in a large retrospectively collected database of sJIA patients with both active sJIA and MAS. As became clear from this review, these diagnostic guidelines are currently the most useful diagnostic tool available. Further improvement in diagnosis will likely come from the addition of promising biomarkers and will allow the early use of targeted therapy.

Since evidence based recommendations on diagnosis and treatment of MAS are still very limited, early expert consultation is recommended as soon as MAS is suspected.
